# In-vivo relation between plasma concentration of sorafenib and its safety in Chinese patients with metastatic renal cell carcinoma: a single-center clinical study

**DOI:** 10.18632/oncotarget.16465

**Published:** 2017-03-22

**Authors:** Haixing Mai, Jun Huang, Yuanyuan Zhang, Nang Qu, Hengyan Qu, Guo-hui Mei, Jiannan Liu, Xiaojie Xu, Lijun Chen

**Affiliations:** ^1^ Department of Urology, 307 Hospital, Affiliated Hospital of Military Medical Sciences, Beijing, China; ^2^ Department of Clinical Pharmacology, 307 Hospital, Affiliated Hospital of Military Medical Science Academy of the PLA, Beijing, China; ^3^ Department of Medical Molecular Biology, Beijing Institute of Biotechnology, Beijing, China

**Keywords:** sorafenib, renal cell cancer, plasma concentration, pharmacokinetics and HPLC-MS/MS

## Abstract

This single-center, observational study analyzed the association between plasma concentration of sorafenib and its safety and efficacy in Chinese patients with metastatic renal cell carcinoma (mRCC). Adult patients with RCC (*n* = 94), treated with sorafenib were enrolled between January 2014 and January 2015. Sorafenib plasma concentrations were measured by liquid chromatography-tandem mass spectrometry. Safety and efficacy variables were evaluated using National Cancer Institute-Common Toxicity Criteria for Adverse Events and Response Evaluation Criteria in Solid Tumors criteria. Association of plasma concentration with safety and efficacy was analyzed. The steady state plasma concentration of sorafenib after 2 weeks of treatment ranged from 881 to 12,526 ng/mL. Major adverse reactions (ADRs) included diarrhea (76.5%), hand-foot syndrome (HFS; 68.99%) and fatigue (55.32%). Significant association was reported between plasma concentration and all the ADRs except rash. At 6 weeks, complete response (CR), partial response (PR), stable disease (SD) and progressive disease (PD) was reported in 3.1%, 13.82%, 52.2% and 13.82% patients, respectively. Objective response and disease control rates were 17.02% and 69.14%. Plasma concentration of sorafenib was >10,000 ng/mL in patients with severe ADRs, which decreased with reduction in dose or discontinuation of treatment. After 21.2 weeks follow-up, median progression free survival was 12.3 months. CR, PR, SD and PD were reported in 1%, 46%, 33% and 19% patients. In conclusion, plasma concentration of sorafenib was associated with its safety and efficacy in Chinese patients with mRCC.

## INTRODUCTION

Advances in molecular research showing aberrant signal transduction activities in renal cell carcinoma (RCC) have paved way to newer targeted drug treatments. These treatments have improved efficacy and tolerability compared with chemotherapy and cytokine therapy with interleukin-2 or interferon-α [1, 2].

Sorafenib tosylate (Nexavar^®^, Bayer Schering Pharma AG, Berlin, Germany, and Onyx Pharmaceuticals, South San Francisco, California, United States), an oral kinase inhibitor demonstrates its anti-proliferative and antiangiogenic properties by targeting RAF, vascular endothelial growth factor receptors and platelet-derived growth factor receptors (PDGFRs) [1, 3, 4]. Sorafenib has been known to significantly improve progression free survival (PFS) and overall survival (OS) [5, 6]. In addition, sorafenib is safe, tolerable [7, 8] and is considered as a standard therapy for metastatic RCC (mRCC) [2]. The Chinese guidelines for RCC treatment recommend sorafenib as the first-line treatment for advanced metastatic RCC (mRCC). It is also recommended as second-line therapy in patients with RCC not responding to 50-mg dose of sunitinib [9].

Safety and efficacy of tyrosine kinase inhibitors (including sorafenib) vary with different ethnicities and drug pharmacokinetics (PK) [10, 11]. PK factors (slow dissolution and enterohepatic circulation) lead to variable absorption and low bioavailability (50%) of sorafenib [12]. Because of these reasons, clinical practitioners prefer an individualized treatment approach instead of the recommended dosage [10, 13]. Sorafenib is reported to be more efficacious in Chinese patients with mRCC compared with the Western counterparts [14, 15]. In addition, the Asians populations (including the Chinese) are more susceptible to adverse events (AEs) of sorafenib [7]. Although effect of genetic variants on efficacy and safety of sorafenib in Chinese patients with mRCC has been determined [16]; no previous study has determined the influence of PK on the safety and efficacy of sorafenib in Chinese patients with mRCC. This study was therefore conducted to explore the association between in-vivo PK characteristics of sorafenib and its safety and efficacy in Chinese patients with mRCC. In addition, the study determined the association between PK and safety in different single nucleotide polymorphism (SNPs) potentially relevant for sorafenib action, metabolism, and transport. This study will provide experimental evidence for personalizing therapy plans in Chinese patients with RCC.

## RESULTS

### Patient demographics and clinical characteristics

A total of 94 patients (age, 21-73 years), whose plasma concentration of sorafenib was monitored during January 2014 to January 2015 at the 307 Hospital, Beijing; China were enrolled in this study. A total of 43 patients had stage 3 and 51 patients had stage 4 RCC at baseline. Of the 94 patients included, 74 patients had received sorafenib 400mg bid, 18 patients received 600mg thrice daily (t.i.d.) and 2 patients received dose of 400mg once daily (q.d.). Other demographic characteristics and concomitant indications of the patients are presented in Table 1. A total of 94 cases of A/A and 2 cases of A/G were reported (Supplementary Table 1).

**Table 1:** Patient demographics & baseline characteristics

	Patients (*N* = 94)
Age (years)	59
Gender Male Female	70 24
ECOG score 0 1 2	43 39 12
Mean duration of using medicine (days) ± SD	449.34 ± 256.0
Mean Concentration (ng/ml) ± SD	4854.83 ± 2899.97
Concomitant indications	
Diarrhea 0 1 2 3	8 30 40 16
HFS 0 1 2 3	12 14 58 10
Alopecia 0 1	66 28
Impairment of liver function 0 1 2	80 6 8
Fatigue 0 1 2	54 12 28
Rash 0 1 2	68 16 10
Hypertension 0 1 2 3	64 26 0 4
Anorexia 0 1	72 22
Leukopenia 0 1	87 7
Skin fading 0 1	90 4
Thrombocytopenia 0 1	90 4
Stage 0 1 2 3 4	0 0 0 43 51

### Sorafenib-related safety outcome

Major ADRs reported with sorafenib treatment included diarrhea (76.50%), hand-foot syndrome (HFS, 66.89%), fatigue (55.2%), hypertension (34.04%) and alopecia (36.17%). The ADRs reported in the study are presented in Table 2. Severe ADRs were reported in 8 patients. In these patients the C_ss_ of sorafenib was > 10,000 ng/mL. Skin fading was observed in 6.38% patients due to a relatively short follow-up after drug administration. Severe ADRs with sorafenib treatment included hypertension (*n* = 2), HFS (*n* = 4) and diarrhea (*n* = 2), which were the primary reasons for treatment discontinuation. Most of the cases of hypertension and HFS were grade 3 and grade 2. A high proportion of patients also reported grade 3 fatigue. All the other ADRs reported in the study were mostly of grade 1, with few grade 2 ADRs. Findings of toxicity analysis are presented in Table 2. All the events were grade 1-3 and no grade 4 toxic effects were observed.

**Table 2:** Safety evaluation

ADR type	No. of cases (%)
ADRs (grade 1 or 2), n (%)	
Diarrhea	72 (76.5)
HFS	64 (68.89)
Alopecia	34 (36.17)
Impairment of liver function	22 (23.40)
Fatigue	52 (55.32)
Rash	28 (29.78)
Hypertension	32 (34.04)
Anorexia	26 (27.66)
Skin fading	6 (6.38)
Toxicities (grade 1-3), n (%)	
Asthenia	67 (71.0)
Mucositis	64 (67.0)
Diarrhea	49 (52.0)
Neutropenia	40 (42.0)
HFS	39 (41.0)

ADR, adverse reaction; HFS, Hand-foot syndrome

### Correlation analysis of the plasma concentration of sorafenib and its side effects

Correlation analysis between ADRs and plasma concentration of sorafenib revealed increased plasma concentration to be significantly associated with the incidence of all the ADRs except rash. The correlation of major ADRs with plasma concentration of sorafenib is presented in Table 3. Severe ADRs were primarily responsible for reduction of sorafenib dose and discontinuation of treatment. In patients with severe ADRs, plasma concentration of sorafenib was > 10,000 ng/mL, which decreased to 5,000-8,000 ng/mL after reducing the dose or discontinuing the treatment. Reduction in dose or discontinuation of drug resulted in resolution of ADRs. ADRs such as HFS, liver function impairment, rash and anorexia were significantly correlated with OS, whereas liver function impairment and anorexia were correlated with PFS, Table 4. The risk of major ADRs in relation with the type of gene polymorphisms is presented in Table 5. It was observed that HF was significantly associated with the genotypes VEGFR2 rs2239702 (OR 6.08, 95% CI 1.08-34.20, *P* = 0.04), ABCB1 rs1045642 (OR 26.35, 95% CI 1.41-490.56, *P* = 0.03), ABCB1 rs2032582 (OR 0.01, 95% CI, 0-0.58, *P* = 0.03), UGT1A1*6 rs4148323 (OR 0.01, 95% CI 0-0.22, *P* = 0.01) and UGT1A9 (OR 105.10, 95% CI, 4.76-2357.78, *P* = 0.01). Fatigue and alopecia were not significantly associated with any of the genetic polymorphism types.

**Table 3:** Correlation between plasma concentration and ADRs

Adverse reactions	*R*	*P*
Fatigue	−0.180	0.022
Diarrhea	0.190	0.020
Impairment of liver function	−0.190	0.042
Rash	0.860	0.330
Anorexia	−0.263	0.004
HFS	0.375	<0.001
Hypertension	0.328	<0.001
Alopecia	0.467	0.004

ADR, adverse reaction

HFS, Hand-foot syndrome

**Table 4:** Correlation of ADRs with OS and PFS

AE	OS		PFS	
	*R*	*P*	*R*	*P*
Diarrhea	0.124	0.120	0.282	0.163
HFS	−0.163	0.040	−0.146	0.467
Alopecia	−0.269	0.226	−0.206	0.313
Impairment of liver function	−0.436	0.040	0.424	0.031
Fatigue	−0.035	0.774	0.103	0.616
Rash	−0.545	0.001	0.235	0.248
Hypertension grades	−0.187	0.262	−0.171	0.402
Anorexia	0.585	0.002	0.570	0.002

**Table 5:** Genetic polymorphism and risk of ADR

	HFS		Hypertension		Alopecia		Fatigue	
	OR	*P*	OR	*P*	OR	*P*	OR	*P*
VEGF rs1570360	0.26(0.04-1.49)	0.13	2.16(0.36-12.86)	0.39	3.48(0.72-16.89)	0.12	0.39(0.09-1.58)	0.39
VEGF rs2010963	0.61(0.14-2.72)	0.52	2.45(0.39-15.55)	0.34	0.45(0.13-1.63)	0.23	1.74(0.57-5.33)	0.33
VEGFR2 rs2239702	6.08(1.08-34.20)	0.04	0.17(0.04-0.74)	0.02	1.78(0.49-6.42)	0.38	0.30(0.06-1.40)	0.13
VEGFR3 rs307826	0.01(0-5.22)	0.14	1.23(0.07-21.98)	0.88	3.16(0.12-86.13)	0.50	0.72(0.04-12.44)	0.82
ABCB1 rs1045642	26.35(1.41-490.56)	0.03	30.40(1.23-751.21)	0.04	0.19(0.01-2.75)	0.22	0.67(0.06-7.14)	0.74
ABCB1 rs1128503	0.37(0.06-2.10)	0.26	8.26(1.49-45.69)	0.02	2.91(0.71-11.87)	0.14	0.32(0.02-5.20)	0.43
ABCB1 rs2032582	0.01(0-0.58)	0.03	0.05(0.01-1.50)	0.08	0.39(0.01-11.16)	0.59	0.61(0.06-6.60)	0.68
CYP3A5 rs776746	0.23(0.02-30.58)	0.56	0.91(0.22-3.85)	0.89	0.93(0.26-3.32)	0.91	1.85(0.54-6.35)	0.33
PDGFR rs1800812	0.09(0.05-1.68)	0.11	20.68(0.87-492.50)	0.06	6.41(0.36-114.65)	0.21	0.61(0.05-8.03)	0.71
UGT1A1*6 rs4148323	0.01(0-0.22)	0.01	6.11(0.70-53.28)	0.10	0.53(0.08-3.45)	0.51	1.29(0.24-6.77)	0.77
UGT1A9	105.10(4.76-2357.78)	0.01	0.30(0.70-1.31)	0.30	1.47(0.38-5.74)	0.58	1.89(0.57-6.21)	0.30

OR, odds ratio

### Efficacy

At the end of 6 weeks, sorafenib treatment resulted in CR, PR and SD in 3 (3.1%), 13 (13.82%) and 49 (52.2%) patients, respectively 6 weeks after initiation of the therapy. PD was reported in 13 patients (13.82%). The ORR (CR + PR) was 17.02% (16/94) and the DCR (CR + PR + SD) was 69.14% (65/94). After a median follow-up of 21.2 months (IQR 8.4-25.6, 95% CI 13.6-28.9), Median PFS was 12.3 months (*n* = 89; IQR 5.8- > 21.2, 95% CI 9.1-15.4) Overall PFS is presented in Figure 2. The PFS was significantly higher in male patients (1) compared with the females (2) (*P* = 0.047, Figure 3). Of the patient population, 62 (70%) were alive by the time of the analysis. ORR was assessed in 78 patients with measurable disease; 1 patient (1%) had a CR, 36 (46%) had a PR. SD and PD were reported in 26 (33%) and 15 (19%) patients. Evaluation of the PFS separately for the stage 3 and stage 4 reported higher PFS in stage 3 patients compared with the stage 4 patients (Figure 4). OS for the whole population is presented in Figure 5 and showed

**Figure 1:**
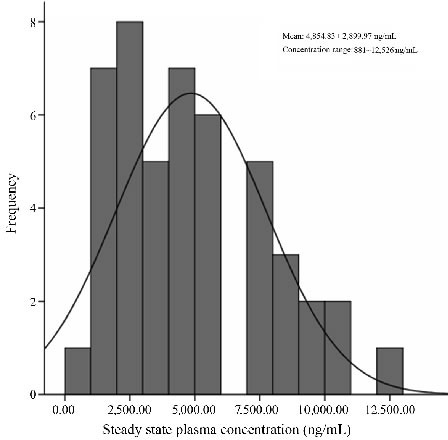
Distribution of plasma concentration

**Figure 2:**
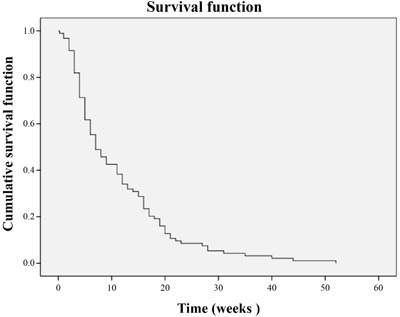
Overall PFS

**Figure 3:**
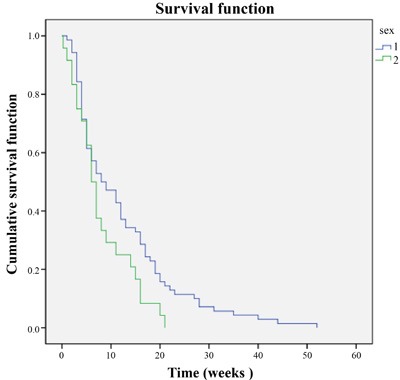
PFS difference among males and females

**Figure 4:**
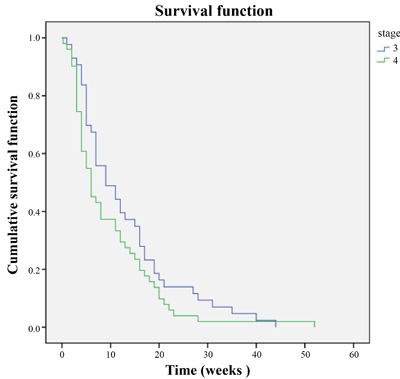
PFS in stage 3 and 4

**Figure 5:**
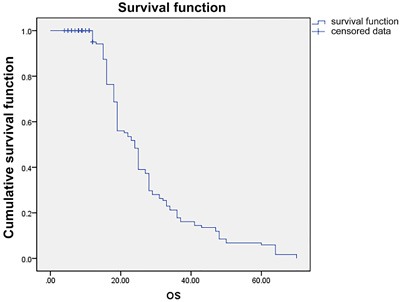
OS in whole population

### Relation between plasma concentration and efficacy of sorafenib

During the study duration, 332 evaluations of plasma concentration of sorafenib were performed in 94 patients. The distribution of C_ss_ of sorafenib is presented in Figure 1. The mean plasma concentration of sorafenib was 4,854.83 ± 2899.97 ng/mL (range, 881-12,526 ng/mL). Plasma concentration was less than the average value in 16 patients; however, it increased more than the average value following administration of a higher dose of sorafenib and this was maintained above the average level by adjusting the administered dose. This resulted in increased compliance and efficacy of the treatment. No significant difference was observed among male and female patients with regard to plasma concentrations of sorafenib (4,501.74 ± 2,664.55ng/mL *vs*. 5,884.68 ± 3,416.11 ng/mL, *P* = 0.294).

## DISCUSSION

To date, there are limited data on the influence of plasma concentration of sorafenib in Chinese patients with RCC. This is one of the very few studies to report dose-dependent safety of sorafenib in China. In this study, sorafenib was primarily associated with ADRs such as diarrhea, fatigue, HFS and hypertension when the plasma concentration was < 10,000 ng/mL. Severe ADRs appeared in a dose-dependent manner, and the incidence of severe diarrhea, HFS and hypertension was reported at a plasma concentration of > 10,000 ng/mL. The ADRs reported in this study are similar to those reported with sorafenib in published literature. Akaza et al. reported elevated lipase (56%), HFSR (55%), rash/desquamation (37%), diarrhea (34%), alopecia (39%), increased amylase (38%) and hypertension (27%) to be the most frequent ADRs in Japanese patients with advanced RCC [17]. Previously published literature also reported increased frequency of hypertension, HFS, fatigue, diarrhea and amylase/lipase levels in patients with advanced RCC [18, 19]. Awada et al reported a dose-dependent increase in the incidence of ADRs, which is in line with the present study [18]. Fukudo et al, also reported a higher concentration of sorafenib in patients with grade ≥ 2 HFS (*P* = 0.0045) and hypertension (*P* = 0.0453) compared with patients without any ADRs [19]. A phase 1 study in patients with RCC reported the highest number of ADRs with the 600-mg bid dose of sorafenib compared with lower doses [20]. In our study, reduction in dose or discontinuation of treatment resulted in resolution of ADRs and increased patient adherence to efficacy of sorafenib. A similar finding was reported in 2 studies in which dose reduction sorafenib resulted in fewer ADRs [21, 22]. The decrease in the incidence of ADRs was possibly due to low plasma concentration of sorafenib. However, Kennoki et al., reported high incidence of ADRs after dose reduction in patients with mRCC who underwent hemodialysis [23]. Published literature suggests that sorafenib is more tolerable among Asian patients with mRCC compared with Western patients. However, there seems a significant difference in the occurrence of ADRs among these populations [14]. The exact reason for these differences are yet to be explored, but, may be relatively smaller body frame and lesser body weight of Asian patients are believed to be the cause for achieving a higher plasma concentration and better efficacy with administration of same dose [14].

The Treatment Approaches in Renal Cancer Global Evaluation Trials demonstrated a significantly higher efficacy of sorafenib compared with placebo in patients with advanced RCC. Patients treated with sorafenib had higher ORR (10% *vs*. 2%) and DCR (80% *vs*. 55%) compared with placebo. The mean PFS and improvement in the quality of life in the sorafenib group was significantly higher compared with placebo (5.8 *vs*. 2.8 months, *P* < 0.01) [5, 6]. A multi-center study in China (*n* = 62) reported CR, PR and SD in 1.75%, 19.3% and 63.16% patients with RCC. The median PFS was 41 weeks, and the DCR and ORR were 84.21% and 21%, respectively [24]. Another study has also demonstrated similar CR, PR, SD and DCR as reported in the present study [25]. In our study, median PFS was 12.3 months, CR, PR, SD and PD was reported in 1%, 46%, 33% and 19% patients, respectively.

In this study, a significant variation in plasma concentration of sorafenib at same dosage (400-600mg) was observed. The oral bioavailability (28-49%), t_1/2_ (25-48 h) and C_ss_ (achieved after 7 days) observed in this study are in line with the prescribing information of sorafenib [3]. The mean plasma concentration was 4,854.83 ± 2899.97 ng/mL and fluctuated between 881 and 12,526 ng/mL, which have not been reported by other researchers. There were 16 patients whose drug concentrations were below the average level. The concentrations were increased above the average value after increasing the drug dosage to 600mg. This study also suggested the effective concentration of sorafenib in Chinese patients to be higher than that reported by other researchers [14]. Because this association could be due to genetic, physiological or pathological factors, the dose and concentration reported in other ethnicities may not be applicable to Chinese patients with RCC. Insufficient or excessive concentration of targeted drugs functioning on their targets may lead to ineffectiveness of treatment or new adverse effects, even drug-induced diseases, therefore effective and safe personalized therapeutic regimen should be tailored according to real-life settings.

AUC curve is the best method to evaluate the blood drug concentration. However, the AUC curve needs to take more time point blood samples and a large number of patients, making it difficult to obtain the total AUC curve. The drug dose repeated constant dose, after 4 to 5 half-lives up to the stable and effective blood drug concentration, the drug absorption rate and elimination rate are balanced, relatively stable blood concentration at a certain level, blood concentration at steady-state plasma concentration, usually expressed in Css (mg/L). Measuring trough concentrations has certain clinical significance linked which include: no effect of absorption and distribution and concentration measured at trough level reflect the drug concentrations mentioned in the published literature. Other advantages of being economical, less time consuming and more practical approach are the reasons we selected measuring trough concentrations instead of AUC [26, 27].

Genetic variation in VEGF, CYP3A and UGTIA leads to altered metabolism of sorafenib and resultant toxicity [15, 25, 28, 29]. In a study by Qin et al, polymorphisms in rs1570360 in VEGF and rs2239702 in VEGFR2 were associated with significant PFS, specifically patients with variant genotypes (AG + AA) polymorphisms both had an unfavorable PFS. Additionally, comparison of patients with rs2239702 GG genotype, patients with the AG + AA genotype demonstrated an unfavorable OS. These findings suggested that VEGF and VEGFR2 showed association with clinical outcomes and toxicity of sorafenib [16]. However, in a case-control study by Sáenz-lópez, VEGF polymorphisms did not exert any significant influence on progression or prognosis of RCC [30]. Expression and levels of PDGFR-α is independently associated with in RCC patients treated with sorafenib, therefore considering expression of PDGFR-α may likely benefit patients with RCC [28]. Rouquette et al demonstrated that patients at high risk for early sorafenib-induced severe toxicity had high cumulated drug exposure and polymorphism of UGTIA9 (rs17868320) [29]. Sorafenib has shown greater efficacy in Chinese patients with mRCC compared with Western population, which might be associated with genetic variation between the 2 populations. However, ADRs such as HFS and alopecia are more commonly observed in Chinese patients than the Western counterparts [31]. In our study, sorafenib treatment resulted in toxicities which included asthenia, mucositis, diarrhea, neutropenia and HFS. As observed in earlier studies, polymorphism in VEGF, CYP3A and UGTIA were associated with different ADRs of sorafenib. In addition, SNP distribution (VEGFR3 (rs307826) and CYP3A5*1 (rs776746)) in Chinese patients with RCC was different from the Western patients, as Chinese patients has higher wild type VEGFR3 (rs307826). Genetic polymorphism of UGT1A1 has been associated with toxicities of sorafenib including elevated bilirubin [32]. Another in-vitro study evaluating the effects of UGT1A variants on PK of sorafenib reported that patients with UGT1A1, UGT1A9, and ABCC2 genotypes had abnormally high area under the curve (AUC) for sorafenib [33]. Hypertension and HFS occurred in high frequency in patients with VEGFR2 rs1870377 variant compared with wild type carriers [34]. In the present study, the genetic variants under evaluation also reported association with safety of sorafenib. Genetic variants VEGFR2 rs2239702, ABCB1 rs1045642 and ABCB1 rs2032582 were significantly associated with increased risk of HFS and hypertension; ABCB1 rs1128503 was reported associated with increased risk of hypertension; UGT1A1*6 rs4148323 and UGT1A9 were associated with hypertension.

Our study has a few limitations due to which the findings must be interpreted with caution. Firstly, the patients were not included randomly and the number of patients included in this single-centre study was small. Secondly, the follow-up period was small. Therefore large-scale, multi-center, randomized studies are needed to validate these findings.

In conclusion, the plasma concentration of orally administered sorafenib was found to be related to its safety in Chinese patients with RCC. In addition, dose-dependent increase in incidence of ADRs was observed with increase in dose of sorafenib. These findings will enable clinical practitioners in China to personalize the dose of sorafenib as per the patient's requirements and minimize the incidents of ADRs.

## MATERIALS AND METHODS

### Study design and patient population

This was a single-center, observational study that enrolled patients with RCC between January 2014 and January 2015. Patients were considered eligible if they had histological confirmation of non-resectable advanced mRCC; aged > 18 years; had Eastern Cooperative Oncology Group (ECOG) performance status grade 0 to 2; life expectancy of at least 12 weeks; and willing to provide informed consent. Patients were considered ineligible for the study if they were unstable or had severe cardiac disease or uncontrolled brain metastases or any other factors regarded as inappropriate for this study by the investigators.

The study protocol was approved by the ethics committee of the 307 Hospital, Beijing, China in accordance with the national and international guidelines and confirmed to Declaration of Helsinki (1964) and its subsequent revisions. All patients received information on the purpose and conduct of this study, and provided written, informed consent.

### Study treatment plan

The patients enrolled in the study had received sorafenib (400 mg) orally twice daily continuously in a 4-week cycle until disease progression, unacceptable toxicities, or mortality. Blood samples were collected on or after 7 days of drug administration. The samples were collected in the morning before administration of drug. For treatment-emergent toxicities of grade 3 or higher, therapy was withheld until resolution to grade 1 or lower. If the patient experienced grade 3 or 4 drug-related toxicity, second time, the dosage was decreased by one level. The dose of sorafenib was escalated to 600 mg in patients who tolerated the standard dose of sorafenib well. Sorafenib was started after a gap of 2 weeks in patients who underwent surgery. The patients were monitored for duration of 3 months. Treatment was continued until tumor progression or occurrence of uncontrolled ADRs.

### Safety and efficacy evaluation

The primary outcome of our study was to determine the association of sorafenib plasma concentration with its safety and efficacy. The secondary outcome included determination of PK parameters in different SNPs and the safety in patients with different SNPs.

Pre-treatment evaluations were conducted 7 days before enrolment. The evaluation included physical examinations (weight, height, vital signs), ECOG performance status, documentation of all measurable or evaluable disease, recording of concurrent medications, routine laboratory evaluations, and pregnancy tests for women. Physical examinations were performed on day 1 of each treatment cycle, hematological laboratory evaluations twice weekly during the treatment period, and biochemical evaluations twice weekly during the first cycle, and twice per cycle thereafter. Clinical tumor measurements were carried out as part of the physical examination.

ADRs were assessed as per the National Cancer Institute-Common Toxicity Criteria for Adverse Events (NCI-CTCAE) Version 3.0 [35]. The toxic AEs were analyzed on the basis of clinical relevance and grading objectiveness, together with grade 3-4 AEs. Efficacy of treatment was evaluated using Response Evaluation Criteria in Solid Tumors criteria, Version 1.1 [36]. We examined tumor responses which include complete response (CR), partial response (PR), stable disease (SD), and progressive disease (PD) rates, as the secondary outcome for the patients treated with sorafenib. Response rate was defined as the proportion of patients with CR and PR in the analyzed population. Patients alive or lost to follow-up were censored at their last follow-up date.

### Analysis of sorafenib concentration

Previous studies have reported attainment of steady state plasma concentration of sorafenib concentration after 7 days of therapy initiation [18, 22]. after the dose, as steady state plasma concentration of sorafenib is achieved Several methods for sorafenib quantification in humans have been published [37–39], using liquid chromatography-tandem mass spectrometry (LC-MS/MS), with lower level of quantification of 7.3 ng/mL (linearity up to 7260 ng/mL) and 5 ng/mL (linearity up to 2000 ng/mL) [38, 39]. Plasma PK parameters - AUC, maximum concentration (C_max_), time to maximum concentration (t_max_), and apparent terminal half-life (t_1/2_) were measured using Agilent G6460 Triple Quadrupole LC/MS System and Agilent Mass Hunter Workstation software version B5.00 (5301 Stevens Creek Blvd. Santa Clara, CA 95051 United States). The PK parameters of sorafenib were analyzed using non-compartmental analysis.

### Single nucleotide polymorphism (SNP) selection

Potentially functional SNP in sorafenib PK genes (CYP3A4, CYP3A5, CYP1A1, CYP1A2, ABCB1, and ABCB2) or sorafenib pharmacodynamics genes (VEGF, VEGFR1, VEGFR2, VEGFR3, PDGFR, PDGFRB, IL8, HIF1A and EPAS1), and sorafenib metabolic genes (UGT1A1*6, UGT1A9) were assessed according to the following criteria: (1) located in the 5′ flanking regions, 5 untranslated region (UTR), 3′ UTR, or coding regions with amino acid changes; (2) minor allele frequency (MAF) > 5% in Chinese population. Finally, 10 polymorphisms associated with PK, pharmacodynamics and metabolism of sorafenib were selected. Three functional SNPs were analyzed. The polymorphisms potentially affecting PK of sorafenib were located in genes for metabolism (CYP3A4 [rs2740574] and CYP3A5 [rs776746]) 11 and transport (ABCB1 [rs1045642, rs1128503, and rs2032582] and ABCG2 [rs221142]) [40]. CYP3A5*1, a high-activity allele is promoter variant with contradictory results published about its activity [41].

### DNA extraction and genotyping

DNA was isolated using the FlexiGene DNA kit (Qiagen, Valencia, CA, USA) and final DNA concentration was quantified with PicoGreen (Invitrogen, Carlsbad, CA, USA). Genotyping of SNPs was done using KASPar SNP genotyping system (Kbiosciences, Hoddesdon, UK). Fluorescence detection and allele assignment were evaluated using detection System 7900HT (Applied Biosystems, Foster City, CA, USA).

The analysis of sorafenib concentration, SNP selection and DNA extraction and genotyping were performed at the Department of clinical pharmacology, at the Affiliated Hospital of Military Medical Science Academy of the PLA, Beijing, China

### Data collection

All the demographic and clinical data were recorded on specific case record forms. The forms were periodically monitored by an external monitor.

### Statistical analysis

The sample size was not defined on the basis of an end point hypothesis, but rather to provide information about the safety and efficacy of the analysis. All the patients who met the inclusion criteria during between January 2014 and January 2015 were included in the study. Continuous data were expressed as mean ± SD or as median, as appropriate, whereas categorical data were summarized as frequencies and percentages. No comparative analyses were performed. A univariate correlation analysis was performed to find the association of plasma concentration and ADRs. Statistical analyses were performed using the SPSS version 22.0 (SAS Corporation, Cary, NC, USA) and Stata version 10 SE (StataCorp, College Station, TX, USA). Safety was analyzed using descriptive methods, in the treated population, according to the treatment received by patients who received at least one dose of sorafenib. Kaplan-Meier method was used to calculate the PFS in enrolled patients.

## References

[1] Hutson E (2011). Targeted therapies for the treatment of metastatic renal cell carcinoma: clinical evidence. Oncologist.

[2] Molina AM, Motzer RJ (2011). Clinical practice guidelines for the treatment of metastatic renal cell carcinoma: today and tomorrow. Oncologist.

[3] Sorafenib prescribing information. http://www.accessdata.fda.gov/drugsatfda_docs/label/2010/021923s008s009lbl.pdf.

[4] Canter RJ, Ames E, Mac S, Grossenbacher SK, Chen M, Li CS, Borys D, Smith RC, Tellez J, Sayers TJ, Monjazeb AM, Murphy WJ (2014). Anti-proliferative but not anti-angiogenic tyrosine kinase inhibitors enrich for cancer stem cells in soft tissue sarcoma. BMC Cancer.

[5] Escudier B, Eisen T, Stadler WM, Szczylik C, Oudard S, Siebels M, Negrier S, Chevreau C, Solska E, Desai AA, Rolland F, Demkow T, Hutson TE (2007). Sorafenib in advanced clear-cell renal-cell carcinoma. N Engl J Med.

[6] Escudier B, Eisen T, Stadler WM, Szczylik C, Oudard S, Staehleret M, Negrier S, Chevreau C, Desai AA, Rolland F, Demkow T, Hutson TE, Gore M (2009). Sorafenib for treatment of renal cell carcinoma: final efficacy and safety results of the phase iii treatment approaches in renal cancer global evaluation trial. J Clin Oncol.

[7] Kim SH, Kim S, Nam BH, Lee SE, Kim CS, Seo IY, Kim TN, Hong SH, Kwon TG, Seo SI, Joo KJ, Song K, Kwak C (2015). Metastatic renal cell carcinoma in Korean patients: results from a retrospective multicenter study. PLoS One.

[8] Park SJ, Lee JL, Park I, Park K, Ahn Y, Ahn JH, Lee DH, Ahn S, Song C, Hong JH, Kim CS, Ahn H (2012). Comparative efficacy of sunitinib versus sorafenib as first line treatment for patients with metastatic renal cell carcinoma. Chemotherapy.

[9] Guo J, Ma J, Sun Y, Qin S, Ye D, Zhou F, He Z, Sheng X, Bi F, Cao D, Chen Y, Huang Y, Liang H (2015). Chinese guidelines on the management of renal cell carcinoma (2015 edition). Ann Transl Med.

[10] Terada T, Noda S, Inui KI (2015). Management of dose variability and side effects for individualized cancer pharmacotherapy with tyrosine kinase inhibitors. Pharmacol Ther.

[11] Zhang H, Dong B, Lu JJ, Yao X, Zhang S, Dai B, Shen Y, Zhu Y, Ye D, Huang Y (2009). Efficacy of sorafenib on metastatic renal cell carcinoma in Asian patients: results from a multicenter study. BMC Cancer.

[12] Di Gion P, Kanefendt F, Lindauer A, Scheffler M, Doroshyenko O, Fuhr U, Wolf J, Jaehde U (2011). Clinical PK of tyrosine kinase inhibitors. Clin Pharmacok.

[13] Al-Rajabi R, Patel S, Ketchum NS, Jaime NA, Lu TW, Pollock BH, Mahalingam D (2015). Comparative dosing and efficacy of sorafenib in hepatocellular cancer patients with varying liver dysfunction. J Gastrointest Oncol.

[14] Ye DW, Zhang HL (2014). Critical appraisal of sorafenib in the treatment of Chinese patients with renal cell carcinoma. Onco Targets Ther.

[15] Yu X, Guo G, Li X, Zhang C, Huang L, Fang D, Song Y, Zhang X, Zhou L (2015). Retrospective analysis of the efficacy and safety of sorafenib in Chinese patients with metastatic renal cell carcinoma and prognostic factors related to overall survival. Medicine.

[16] Qin C, Cao Q, Li P, Wang S, Wang J, Wang M, Chu H, Zhou L, Li X, Ye D, Zhang H, Huang Y, Dong B (2016). The influence of genetic variants of sorafenib on clinical outcomes and toxic effects in patients with advanced renal cell carcinoma. Scien Rep.

[17] Akaza H, Tsukamoto T, Murai M, Nakajima K, Naito S (2007). Phase II study to investigate the efficacy, safety, and PK of sorafenib in Japanese patients with advanced renal cell carcinoma. Jpn J Clin Oncol.

[18] Awada A, Hendlisz A, Gil T, Bartholomeus S, Mano M, de Valeriola D, Strumberg D, Brendel E, Haase CG, Schwartz B, Piccart M (2005). Phase I safety and PK of BAY 43-9006 administered for 21 days on/7 days off in patients with advanced, refractory solid tumours. Br J Cancer.

[19] Fukudo M, Takuma I, Mizuno T, Shinsako K, Hatano E, Uemoto S, Kamba T, Yamasaki T, Ogawa O, Seno H, Chiba T, Matsubara K (2014). Exposure-toxicity relationship of sorafenib in japanese patients with renal cell carcinoma and hepatocellular carcinoma. Clin Pharmacok.

[20] Moore M, Hirte HW, Siu L, Oza A, Hotte SJ, Petrenciuc O, Cihon F, Lathia C, Schwartz B (2005). Phase I study to determine the safety and PK of the novel RAF kinase and VEGFR inhibitor BAY 43-9006, administered for 28 days on/7 days off in patients with advanced, refractory solid tumors. Ann Oncol.

[21] Yang L, Shi L, Fu Q, Xiong H, Zhang M, Yu S (2012). Efficacy and safety of sorafenib in advanced renal cell carcinoma patients: Results from a long-term study. Oncol Lett.

[22] Strumberg D, Clark JW, Awada A, Moore MJ, Richly H, Hendlisz A, Hendlisz A, Hirte HW, Eder JP, Lenz HJ, Schwartz B (2007). Safety, PK, and preliminary antitumor activity of sorafenib: a review of four phase I trials in patients with advanced refractory solid tumors. Oncologist.

[23] Kennoki T, Kondo T, Kimata N, Murakami J, Ishimori I, Nakazawa H, Hashimoto Y, Kobayashi H, Iizuka J, Takagi T, Yoshida K, Tanabe K (2011). Clinical results and PK of sorafenib in chronic hemodialysis patients with metastatic renal cell carcinoma in a single center. Jpn J Clin Oncol.

[24] Sun Y, Na Y, Yu S, Zhang Y, Zhou A, Li N, Yang L, Lou G (2008). Sorafenib in the treatment of Chinese patients with advanced renal cell cancer. J Clin Oncol.

[25] Maroto P, Rini B (2014). Molecular Biomarkers in Advanced Renal Cell Carcinoma. Clin Cancer Res.

[26] Maganti L, Panebianco DL, Maes AL (2008). Evaluation of Methods for Estimating Time to Steady State with Examples from Phase 1 Studies. AAPS J.

[27] Kang JS, Lee MH (2009). Overview of Therapeutic Drug Monitoring. Korean J Intern Med.

[28] Kusuda Y, Miyake H, Behnsawy HM, Fukuhara T, Inoue TA, Fujisawa M (2013). Prognostic prediction in patients with metastatic renal cell carcinoma treated with sorafenib based on expression levels of potential molecular markers in radical nephrectomy specimens. Urol Oncol.

[29] Rouquette PB, Narjoz C, Golmard JL, Schoemann AT, Mir O, Taieb F, Durand JP, Coriat R, Dauphin A, Vidal M, Tod M, Loriot MA, Goldwasser F (2012). Early Sorafenib-Induced Toxicity Is Associated with Drug Exposure and UGTIA9 Genetic Polymorphism in Patients with Solid Tumors: A Preliminary Study. PlosOne.

[30] Sáenz-López P, Vazquez F, Cozar JM, Carretero R, Garrido F, Ruiz Cabello F (2013). VEGF polymorphisms are not associated with an increased risk of developing renal cell carcinoma in Spanish population. Hum Immunol.

[31] Ye DW, Zhang HL (2014). Critical appraisal of sorafenib in the treatment of Chinese patients with renal cell carcinoma. Onco Targets Ther.

[32] MezaJunco J, Chu QS, Christensen O, Rajagopalan P, Das S, Stefanyschyn R, Sawyer MB (2009). UGT1A1 polymorphism and hyperbilirubinemia in a patient who received sorafenib. Cancer Chemother Pharmacol.

[33] Peer CJ, Sissung TM, Kim A, Jain L, Woo S, Gardner ER, Kirkland CT, Troutman SM, English BC, Richardson ED, Federspiel J, Venzon D, Dahut W (2012). Sorafenib is an inhibitor of UGT1A1 but is metabolized by UGT1A9: implications of genetic variants on PK and hyperbilirubinemia. Clin Cancer Res.

[34] Jain L, Sissung TM, Danesi R, Kohn EC, Dahut WL, Kumar S, Venzon D, Liewehr D, English BC, Baum CE, Yarchoan R, Giaccone G, Venitz J (2010). Hypertension and hand-foot skin reactions related to VEGFR2 genotype and improved clinical outcome following bevacizumab and sorafenib. J Exp Clin Cancer Res.

[35] Common Terminology Criteria for Adverse Events v3.0 (CTCAE). http://ctep.cancer.gov/protocolDevelopment/electronic_applications/docs/ctcaev3.pdf.

[36] Eisenhauer EA, Therasse P, Bogaerts J, Schwartz LH, Sargent D, Ford R, Dancey J, Arbuck S, Gwyther S, Mooney M, Rubinstein L, Shankar L, Dodd L (2009). New response evaluation criteria in solid tumours: revised RECIST guideline (version 1.1). Eur J Cancer.

[37] Zhao M, Rudek MA, He P, Hafner FT, Radtke M, Wright JJ, Smith BD, Messersmith WA, Hidalgo M, Baker SD (2007). A rapid and sensitive method for determination of sorafenib in human plasma using a liquid chromatography/tandem mass spectrometry assay. J Chromatogr B Analyt Technol Biomed Life Sci.

[38] Jain L, Gardner ER, Venitz J, Dahut W, Figg WD (2008). Development of a rapid and sensitive LCMS/MS assay for the determination of sorafenib in human plasma. J Pharm Biomed Anal.

[39] Jain L, Gardner ER, Figg WD, Chernick MS, Kong HH (2010). Lack of association between excretion of sorafenib in sweat and hand-foot skin reaction. Pharmacotherapy.

[40] Williams JA, Ring BJ, Cantrell VE, Jones DR, Eckstein J, Ruterbories K, Hamman MA, Hall SD, Wrighton SA (2002). Comparative metabolic capabilities of CYP3A4, CYP3A5, and CYP3A7. Drug Metab Dispos.

[41] Kuehl P, Zhang J, Lin Y, Lamba J, Assem M, Schuetz J, Watkins PB, Watkins PB, Daly A, Wrighton SA, Hall SD, Maurel P, Relling M (2001). Sequence diversity in CYP3A promoters and characterization of the genetic basis of polymorphic CYP3A5 expression. Nat Genet.

